# Photo- and Water-Degradation Phenomena of ZnO Bio-Blend Based on Poly(lactic acid) and Polyamide 11

**DOI:** 10.3390/polym15061434

**Published:** 2023-03-14

**Authors:** Roberta Puglisi, Andrea Antonino Scamporrino, Nadka Tzankova Dintcheva, Giovanni Filippone, Elena Bruno, Paola Scarfato, Pierfrancesco Cerruti, Sabrina Carola Carroccio

**Affiliations:** 1 Institute for Polymers, Composites and Biomaterials IPCB-CNR, Via P. Gaifami 18, 95126 Catania, Italy; 2 Dipartimento di Ingegneria, Università di Palermo, Viale delle Scienze, ed. 6, 90128 Palermo, Italy; 3 Department of Chemical, Materials and Production Engineering, University of Naples Federico II, Piazzale V. Tecchio 80, 80125 Naples, Italy; 4 Department of Physics and Astronomy “E. Majorana”, University of Catania, Via S. Sofia 64, 95123 Catania, Italy; 5 Institute for Microelectronics and Microsystems IMM-CNR, Via S. Sofia 64, 95123 Catania, Italy; 6 Department of Industrial Engineering, University of Salerno, Via Giovanni Paolo II, 84084 Fisciano, Italy; 7 Institute for Polymers, Composites and Biomaterials (IPCB-CNR), Via Campi Flegrei 34, 80078 Pozzuoli, Italy

**Keywords:** PLA, PA11, biopolymer nanocomposite, ZnO nanofiller, hydrolytic degradation, photo-oxidation

## Abstract

The goal of this work was to investigate the morphological and chemical–physical changes induced by adding ZnO nanoparticles to bio-based polymeric materials based on polylactic acid (PLA) and polyamide 11 (PA11). Precisely, the photo- and water-degradation phenomena of nanocomposite materials were monitored. For this purpose, the formulation and characterization of novel bio-nanocomposite blends based on PLA and PA11 at a ratio of 70/30 wt.% filled with zinc oxide (ZnO) nanostructures at different percentages were performed. The effect of ZnO nanoparticles (≤2 wt.%) within the blends was thoroughly explored by employing thermogravimetry (TGA), size exclusion chromatography (SEC), matrix-assisted laser desorption ionization–time-of-flight mass spectrometry (MALDI-TOF MS) and scanning and transmission electron microscopy (SEM and TEM). Adding up to 1% wt. of ZnO resulted in a higher thermal stability of the PA11/PLA blends, with a decrement lower than 8% in terms of molar masses (MMs) values being obtained during blend processing at 200 °C. ZnO promoted trans-ester-amide reactions between the two polymers, leading to the formation of PLA/PA11 copolymers. These species could work as compatibilisers at the polymer interface, improving thermal and mechanical properties. However, the addition of higher quantities of ZnO affected such properties, influencing the photo-oxidative behaviour and thus thwarting the material’s application for packaging use. The PLA and blend formulations were subjected to natural aging in seawater for two weeks under natural light exposure. The 0.5% wt. ZnO sample induced polymer degradation with a decrease of 34% in the MMs compared to the neat samples.

## 1. Introduction

In the last decade, the urgent need to replace traditional polymeric materials with sustainable ones has boosted research toward ad hoc biopolymer blending formulations. To merge the single properties of each polymeric component and to obtain materials with the particular advantages of traditional plastics, great attention has been focused on binary bio blends formed by poly(lactic acid) (PLA) and polyamide 11 (PA 11). Indeed, PLA, one of the favourite biodegradable candidates for replacing petroleum-based polymers, presents a low impact strength and heat distortion temperature (HDT), limiting its application in several fields. Conversely, PA11, produced from castor plants, is characterised by high thermal stability and impact resistance, abrasion, and chemical resistance. Although the two polymers present a weak interfacial interaction [1,2,3,4,5], the resulting blend is poorly miscible, forming a two-phase metastable system that partially thwarts all the expected features [6,7]. To date, remarkable efforts have been made to enhance the compatibility of PLA/PA11 blends using low-cost and sustainable procedures. Among these, the addition of crosslinking agents [8] or nanofillers [3,4,9,10] upon extrusion has been investigated in depth to increase blend miscibility. Notably, the incorporation of nanofillers represents an attractive alternative, providing additional benefits such as an improvement in the thermal stability of the formulated materials [9,11,12]. Furthermore, morphological parameters can remarkably influence the final properties of polymeric blends [13,14]. For instance, the addition of 3% wt. of organoclay (OMMT) to a PLA/PA11 blend, as reported by Nuzzo et al., was demonstrated to change the morphology of the blend, inducing a significant increase in the glass transition temperature of about 100 °C as compared to neat PLA, thereby extending its use for heat-resistant applications [11,15]. Halloysite nanotubes (HNT) strongly influence the morphological and mechanical features of PLA/PA11(80/20) blends [16]. As an example, the literature reports on how the addition of PA11 and HNTs significantly improve the toughness and impact strength of PLA. However, the addition of such nanofillers, even in low amounts, to PLA/PA11-based polymeric blends drastically affects the photostability of both polymeric matrices, especially as far as the PA11 component is involved [17,18,19,20,21,22,23]. Other research groups have reported on the inclusion of cellulose nano crystals (CNCs) via a combination of procedures involving solvent dissolution, casting and melt mixing [4]. They showed that CNCs preferably localised in the PA11 phase without changing the PLA/PA11 morphology for all the compositions tested. Nevertheless, in the co-continuous 50/50 PLA/PA11 blends, the morphology was noticeably affected by the presence of CNCs, reducing the droplet size by suppressing the coalescence. Low-cost and eco-friendly ZnO nanostructures are often used as fillers of polymeric matrices to provide benefits such as thermal stability, gas barrier effects, mechanical properties and antioxidant activity [24,25,26,27]. In addition, ZnO can participate as an active part, introducing UV light barrier and antimicrobial features [27,28,29], catalytic properties [30] and biomedical properties [31,32]. Depending on the distribution of ZnO nanoparticles in the polymer matrix, these nanofillers can work as pro-degradants favouring a reduction in MMs or as protecting agents if layered on the top of the polymer surface [33,34]. However, the effect of ZnO nanofillers on the chemical–physical properties and morphology of PLA/PA11 blends has never been explored in detail, i.e., considering all the chemical and morphological implications related to the addition of nanoparticles within the blend. In this view, the main objective of this work was to perform a thorough study of the effects induced by the addition of ZnO nanoparticles at 0.5%, 1% and 2% wt concentrations on PLA/PA11 blends. Specifically, we focused our investigation on the ZnO-induced effects towards (i) photodegradation behaviour under UV irradiation and (ii) polymer blend degradation in water. Different experimental techniques were employed to shed light on the complex chemical and physical modifications due to the nanofiller [16]. The thermal stability of the single polymers and the new nanocomposites was evaluated through TGA analysis. Size exclusion chromatography (SEC) and MALDI-TOF mass spectrometry measurements were exploited to study the chemical changes in the bulk of the materials as a function of the ZnO content and the aging process. Morphology investigations by TEM and SEM were also performed to reveal the distribution of the nanoparticles in the blends, which is relevant to correctly understanding (and possibly controlling) the degradation pathways associated with the filler. Finally, the effect of immersion in artificial seawater was also investigated for both the PLA reference and the blend formulation. The results obtained are herein reported.

## 2. Materials and Methods

### 2.1. Materials

PLA, D,L racemic mixture Polylactid acid (PLA) Mw 18,000–24,000, 3 mm PA11 pellets, zinc oxide nano powder (<100 nm particles size), trans-2-[3-(4-tert-Butylphenyl)-2-methyl-2-propenylidene]malononitrile (DCTB), 1,1,1,3,3,3-hexafluoro-2-propanol (HFIP) and tetrahydrofuran (THF) were purchased from Sigma Aldrich and used with any further purification. The size and morphology of the commercial ZnO filler were analysed by SEM (Figure S1a,b). Nanoparticle sizes spanned a 100–200 nm range, combining tubular and round-shaped forms.

### 2.2. Blending Procedure

To reduce the moisture content whilst avoiding hydrolytic degradation during melt processing, both PLA and PA11 were vacuum-dried at 80 °C for 24 h before processing. PLA/PA11 blends containing varying amounts (0.5, 1, and 2% wt.) of commercial ZnO were prepared by melt mixing using a Brabender PLA-330 internal mixer at 200 °C for 5 min at 50 rpm. To improve the ZnO dispersion in the blend melt, the PLA/PA11 samples were pre-mixed for 1 min. Then, ZnO was added, and the mixing continued for 4 min. Films were obtained by the hot press-forming process.

### 2.3. Size Exclusion Chromatography (SEC)

An SEC system with four Phenogel 5 um 500 A- 300 × 4.6 mm columns arranged in series was used. The analyses were performed at R.T. using 1,1,1,3,3,3-hexafluoro-2-propanol (HFIP) as an eluent with a 0.4 mL/min flow rate. Films of PLA, PA11, blends and nanocomposites were dissolved in HFIP and THF (depending on the sample analysed) with a concentration of 3 mg/mL, and the resulting solutions were filtered before the measurements. A Waters 2414 device was used as a refractive index detector.

### 2.4. Matrix-Assisted Laser Desorption Ionization–Time-of-Flight Mass Spectrometry (MALDI-TOF MS)

MALDI-TOF mass spectra were recorded working in the positive ion mode with a 4800 Proteomic Analyzer (Applied Biosystems, Bedford, MA, USA) MALDI-TOF/TOF instrument equipped with an Nd:YAG laser at a wavelength of 355 nm with a <500 ps pulse and a 200 Hz firing rate operating in the reflectron mode. The accelerating voltage was 15 kV. The detection of the ions was performed in the reflector mode. Samples were dissolved in HFIP with a concentration of 3 mg/mL and mixed with a solution containing trans-2-[3-(4-tert-butylphenyl)-2-methyl-2-propenylidene] malononitrile (DCTB) as the matrix (3 mg/mL in HFIP) to obtain a mixture with a 3:1 weight ratio.

### 2.5. Thermogravimetric Analysis (TGA)

Thermogravimetric analyses of the PLA/PA11 blends and nanocomposites were carried out using a TA Instruments Q500 thermobalance (New Castle, DE, US) with a thermal ramp of 10 °C min^−1^ from 20 °C to 700 °C under a nitrogen flow (60 mL min^−1^).

### 2.6. Scanning Electron Microscopy (SEM)

The morphologies of the blend nanocomposites were investigated by scanning electron microscopy images (SEM, Gemini 152 field emission SEM Supra 25, Carl Zeiss, Oberkochen, Germany). The images were obtained in the Inlens mode at 5 kV. The samples of PLA/PA11 (films and pellets) were cryogenically fractured in liquid nitrogen to obtain a cross section and subsequently sputter-coated with gold (10 mA, 4 min) to create a conductive surface layer of 10 nm.

### 2.7. Transmission Electron Microscopy (TEM)

TEM analysis was used to investigate the localization of ZnO particles in the polymer blend. To this aim, ultrathin sections of the composite blend samples were prepared with a Leica UC7 ultramicrotome system operating at room temperature. The sections were then placed on copper grids, and TEM observations were performed in the bright field mode by an FEI Tecnai G12 Spirit Twin TEM operating at a 120 kV acceleration voltage.

### 2.8. Aging Tests

All samples in film form (1 × 1 cm) with a thickness of 32 ± 3 were subjected to accelerated photo-oxidation by exposing them for up to 366 h with a QUV apparatus equipped with 340 UV lamp at 60 °C. Another set of samples were placed in vials containing artificial seawater (ASW, Instant Ocean Aquarium System, Blacksburg, VA, USA) for two weeks and exposed to natural light at room temperature. Degraded samples were analysed by using SEC and MALDI-TOF MS measurements.

## 3. Results and Discussion

Based on the data reported in the literature [15,35], we selected 70/30 as the target weight % of the PLA/PA11 samples. Indeed, this blend percentage was reported as the most effective in terms of the mechanical properties. The pristine **PLA/PA11** blend was prepared by melting extrusion of neat PLA at 70% wt and PA11 at 30% wt. Since we aimed to exploit the uneven distribution of the nanoparticles that typically occurs in immiscible polymer blends, we decided to deal with a low filler and not to saturate the preferred phase/interphase. Three different nanocomposites, **PLA/PA11@ZnO_0.5**, **PLA/PA11@ZnO_1** and **PLA/PA11@ZnO_2**, were obtained by adding 0.5%, 1% and 2% wt concentrations of ZnO nanoparticles to the polymer mixture during the melt extrusion of the materials, which was performed at 200 °C. The PLA/ZnO and PA11/ZnO nanocomposites were also prepared and tested as references to gain evidence about the possible effect of such nanoparticles on the chemical stability of the selected polymers. The characterization of all the materials was carried out as follows.

### 3.1. TGA Measurements

All the processed samples were characterised by TGA in nitrogen, and the recorded data are reported in Table 1. The neat PLA and PA11 samples degraded at 377 and 425 °C, respectively. As shown in Table 1, adding small amounts of ZnO increased the thermal stability of PA11, whereas higher amounts of filler resulted in a drop-down of the degradation temperatures as expected [30]. Differently, adding ZnO to PLA induced thermal degradation phenomena, as revealed from the relative thermograms (Table 1). It is reasonable to hypothesise that at high temperatures, the ZnO nanoparticles promoted chain cleavage, thereby decreasing the thermal stability of the PLA material [36]. Very interestingly, in the case of the PLA/PA11 nanocomposite blends, the presence of PA11, although as a minor component, induced an increase in the thermal stability compared to the PLA up to a 1% addition of ZnO. This result may suggest that ZnO could be localised in the polyamide phase, protecting the PLA from direct contact with the degrading agent. Such a shielding effect vanished at a 2% addition of ZnO. This could be due to the saturation of the PA11 domains and the consequent exposure of the nanoparticles towards the PLA, which triggered the degradation of the polymer.

### 3.2. SEC Analysis

**Figure 1:**
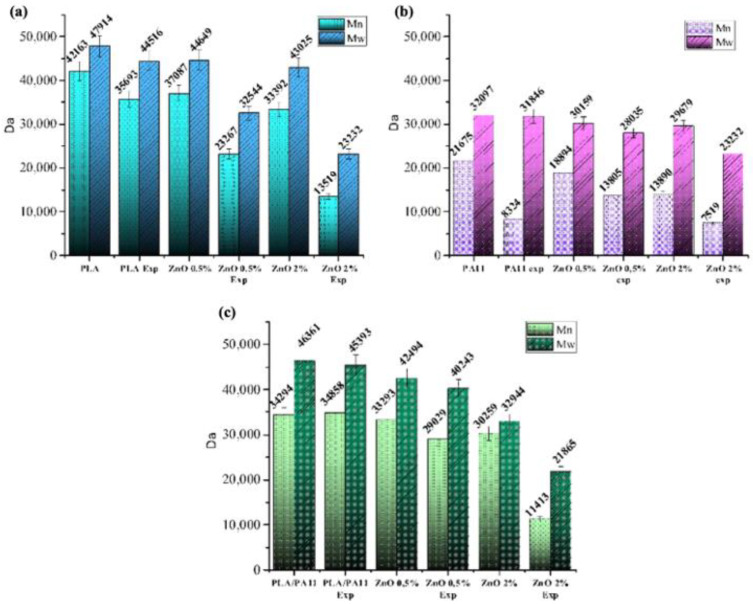
(**a**–**c**) Comparison of the average molecular weight number (Mn) and the weight-averaged molecular weight (Mw) of the PLA (**a**), PA11 (**b**) and PLA/PA11 (**c**) and of their nanocomposites at different contents (weight %) of ZnO nanoparticles after UV exposure at 60 °C and 366 h.

Figure 1a–c report the MMs values collected for all the samples as determined by SEC analysis by using HFIP as the eluent phase (Figure 1a,b).

Since the SEC method provides relative values depending on the hydrodynamic volumes of the standards used, the trend of MMs as a function of the ZnO addition in the PLA/PA11 blends was challenging to assess. Indeed, the presence of two different macromolecular chains complicated the measurements. In addition, the possible formation of a PLA/PA11 block co-polymer during the extrusion process implies the presence of new species with different conformations and, thus, different hydrodynamic volumes. As a result, we could only observe the apparent decrement of the Mw values (Figure 1a–c) as a function of the ZnO content. Significantly, variations in the Mw values were shown by the samples with a ZnO content of 1 and 2%.

By considering the data obtained from the PLA and PA11 samples, we could ascribe the remarkable MW decrements to the reduction in the chains lengths as well as the structural conformation changes in the macromolecules; it is reasonable to hypothesise that the ZnO addition catalysed the ester amide transesterification reaction, forming a new species characterised by specific hydrodynamic volumes not comparable with those of the used standards.

This supposition was confirmed by the other chemical and morphological characterizations discussed in the next sections.

The SEC data collected for all the samples after UV exposure confirmed a significant influence of the filler amount on the reduction in MMs. As expected, the PA11 samples were significantly affected by the light exposure; adding ZnO promoted the photodegradation reactions by further decreasing the MM values. As revealed by other studies [37], PLA manifested a good resistance to UV light up to 366 h, especially compared to PA11. However, the addition of filler at any percentage caused a sensible decrement of the MMs depending on the exposure time. The degradation process was more marked for the samples with higher percentages of ZnO, and this mainly occured due to cleavage of the macromolecular chains, as revealed by SEC. This result agreed with the data from Therias et al. [38]. Similar results were achieved by irradiating the PLA/PA11 blends (Figure 1a–c).

Several papers have reported that the addition of TiO_2_ in an anatase form promotes the photocatalytic degradation of PLA [39]. The ZnO used in this work was based on wurtzite, whose crystalline structure is similar to anatase and possesses a comparable photocatalytic activity. Accordingly, a comprehensive study on the photochemical behaviors of PLA/ZnO nanocomposite films reported by S. Therias et al. confirmed that ZnO being well embedded into polymer matrices induces chain cleavage with a depletion of MM values [38]. Although not desirable from the mechanical properties side, if opportunely tuned, this effect could be exploited to increase the degradability of PLA-based packaging in water mediums.

To this purpose, a preliminary investigation was performed by immersing the PLA and PLA/PA11 composites in simulated seawater and exposing them to natural sunlight for two weeks at room temperature and under shaking. The SEC profiles collected using a THF solvent were reported as a function of the ZnO content (see Supplementary Figures S27 and S28). The PLA@ZnO and PLA/PA11@ZnO degraded faster than the unfilled references [40]. Notably, the MM decrement was remarkable for the sample with a 0.5% filler addition (Figures S27 and S28, Table 2 and Table 3). As reported in Table 1, this amount increased the thermal stability of the nanocomposites without influencing the molecular parameters for both the PLA and PLA/PA11 formulations. In terms of adding value, the water degradation of the PLA and PA11 components may be nicely increased. This property could make a difference from an environmental point of view. Indeed, the degradation of PLA in seawater is a time-demanding process at room temperature [40].

### 3.3. MALDI Mass Spectrometry Measurements

The high sensitivity of MALDI-TOF MS was mainly used to observe the macromolecular structures forming the polymeric chains. Specifically, the end group determination obtained by MALDI can give detailed data on the polymeric changes induced by temperature, oxygen [41], light and mechanical stresses in the presence or absence of nanofillers, recognizing the first stage of the degradation phenomena, which is not possible using other techniques. In this view, the MALDI-TOF spectra of each polymer processed by adding different contents of ZnO nanofiller were obtained and are reported in the supplementary information (Figures S2–S19) as well as the table containing peaks assignments (Table S1). The spectrum of the neat PLA processed at 200 °C revealed that the most abundant species at higher MM levels consisted of acid/alcoholic terminal groups. At the lower mass range (enlarged portion of PLA MALDI spectrum at 1450–1710 *m*/*z*), cyclic oligomers were present (Figure S11). The processing of PLA by adding ZnO nanoparticles at different percentages produced limited degradation events, as suggested by comparing the peaks with acid/alcoholic terminal groups in the low-mass region of the spectra (Figures S12–S17). Specifically, the hydrolysis process occurring along the PLA chains slightly reduced the MMs, as was also revealed from the SEC data reported above.

Regarding the PA11 sample (Supplementary Figure S3), the peak ascribed to the linear specie NH_2_/COOH 1484.56 (Table S1, structure 4) was comparable in intensity with the peak at *m*/*z* 1488,59. The latter could bear a double contribution derived from the cyclic specie and the thermal degradation product (Table S1) [42,43]. Upon increasing the ZnO content (Figures S5, S7 and S9), the relative intensity of the peak at *m*/*z* 1488.59 strongly decreased. This result may suggest an inhibition of the thermal degradation process, probably due to the presence of ZnO. This hypothesis was supported by thermogravimetric analysis, which showed the increased thermal stability of the PA11 containing ZnO (Table 1).

**Figure 2:**
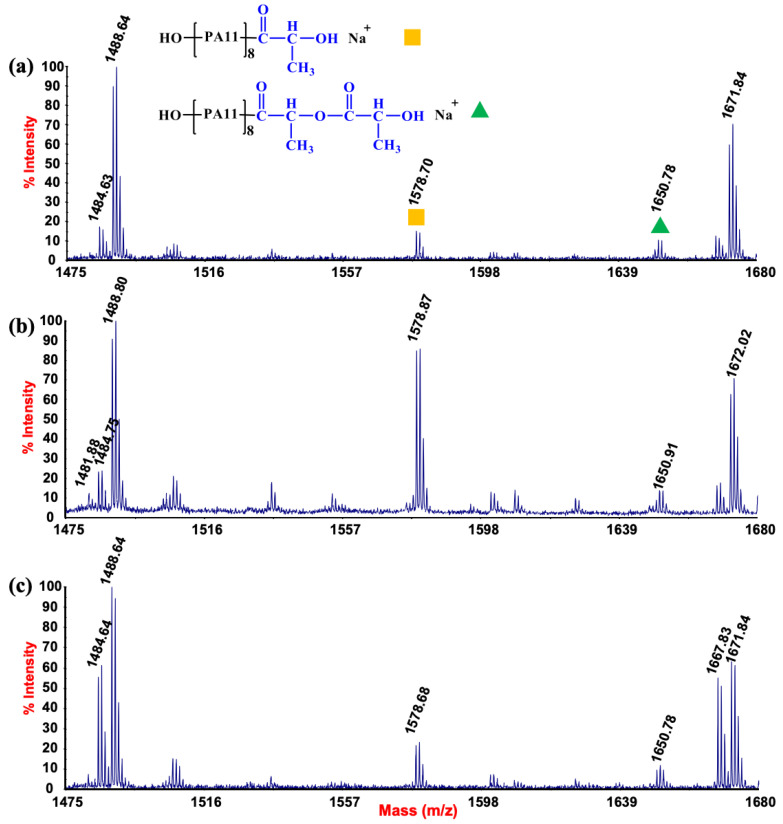
(**a**–**c**) Comparison of MALDI-TOF spectra of (**a**) PLA/PA11, (**b**) PLA/PA11@ZnO_0.5 and (**c**) PLA/PA11@ZnO_2 in the *m*/*z* range 1475–1680.

The trend of the intensities of the characteristic MALDI peaks of the PLA/PA11 and the related ZnO nanocomposites is shown in Figure 2a–c. The spectrum of the PLA/PA11 samples presented both particular PLA and PA11 peaks, as assigned in Table S1. Specifically, the peaks at 1466.64 and 1488.64 *m*/*z* belonged to cyclic PA11 macromolecular adducts, whereas the peaks at 1484.64 and 1506.64 *m*/*z* were attributed to linear terminated NH_2_/COOH chains. The peaks related to PLA (*m*/*z* 1463.75 and *m*/*z* 1481.76) appeared much less intense compared to the PA11 signals, although the percentage of PLA in the blend was higher than PA11. This fact depended on the fact that PA11 macromolecules are capable of desorbing and ionizing under a large range of MALDI conditions and parameters. Thus, the PA11 oligomers were preferentially detected, suppressing the PLA signals.

Interestingly, new peaks with a low relative abundance at 1578.68 and 1650.78 *m*/*z* appeared in the spectrum. The latter was unambiguously assigned to co-block linear PLA/PA11 chains with COOH/OH terminal groups (Figure 2). Figure 2b shows the MALDI spectra of the blend collected after adding 0.5% ZnO, where these adducts became predominant among the MALDI signals. The appearance of these peaks proved that ester–amide reactions were already activated during the hot melting stage, although the addition of ZnO nanoparticles seemed to help the kinetics of the process. In addition, a slight increment of the peak intensities belonging to linear PLA chains may indicate the initial occurrence of the hydrolysis process. A higher number of acid terminal groups in the blend could reasonably increase the trans-ester–amide reactions. Nevertheless, as revealed from spectra collected for the PLA/PA11@ZnO_2% samples, the associated peak intensity decreased. This phenomenon was ascribed to the formation of a higher amount of PA11 linear chain oligomers that could suppress the signals of copolymers.

The photo-degradation of PLA, PA11 and the relative 70/30 blend in the presence of ZnO nanoparticles were also analysed by MALDI measurements.

The photo-oxidation processes caused by the UV exposure of both the neat polymers are well known [17,19,38,44,45]. PA11 significantly photodegrades in the presence of oxygen by αH-abstraction and Norrish I and II photoreactions. Conversely, PLA manifests excellent durability while irradiated, degrading at a higher exposure time than aliphatic polyamides.

As reported in the literature, PLA photodegradation involves similar cleavage mechanisms to those reported for PA11 [46]. The presence of ZnO generates additional degradation routes caused by reactive oxygen species (ROS). ROS, which are formed by the photocatalytic conversion of oxygen or water moisture, can generate a radical attack on the polymer chains without preferential cleavages. After 366 h of UV exposure, the MALDI spectrum of the PLA charged with 0.5% ZnO displayed the appearance of several peaks that were reasonably attributed to random C-C breaking along with the aliphatic chains (see Figure S21) [39].

In addition, for the PA11 samples, ZnO promoted earlier degradation processes, whose mechanisms partially differ from the well-stated photo-oxidative reactions. Similarly to the PLA, new peaks appeared in the spectra of the photo-oxidised PA11@ZnO samples compared to those of the PA11 photo-oxidised samples [14]. The latter were produced at a significantly lower UV exposure time (168 h) than the PLA 0.5% sample. As visible from the MALDI spectrum reported in Figure S20, the distances between two proximal signals were 14 uma, denoting an appropriate degradation level of the material [30].

### 3.4. SEM and TEM Analyses

**Figure 3:**
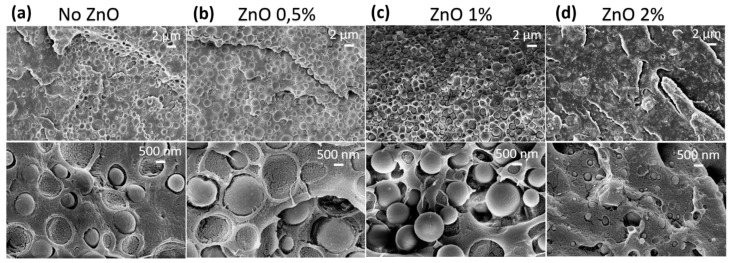
Cross-section SEM images of (**a**) PLA/PA11 70/30 pristine film, (**b**) PLA/PA11 70/30 + ZnO 0.5%, (**c**) PLA/PA11 70/30 + 1% ZnO and (**d**) PLA/PA11 70/30 + 2% ZnO.

The morphological investigation was performed by SEM and TEM analysis of the blend samples with and without the addition of ZnO nanoparticles. Figure 3 displays the SEM characterization of the samples. All the images show a typical two-phase morphology, where the minor phase (PA11) is dispersed in the major one (PLA) with a nodular structure. As revealed from Figure 3a–d, the size of the PA11 droplets decreased with increasing the amount of ZnO added, indicating increased compatibility. The two phases were no longer distinguishable for the PLA/PA11@ZnO 2% sample. The mean size and distribution of the PA11 droplets are reported in Table S2 and Figure S21. The decrease was observed to range from 1.02 μm for the PLA/PA11 sample to 1.43 μm for the PLA/PA11@ZnO_1 sample. This evidence was likely due to the trans-ester–amide reactions occurring at the molecular level, and it was also accompanied by degradation phenomena, which were no longer negligible at a higher ZnO content. This trend nicely supports the data obtained from the SEC analysis. Indeed, Mw depletion was associated with the change in the macromolecular chain length or the configuration, whereas the MALDI data confirmed the formation of PLA-PA11 copolymers.

**Figure 4:**
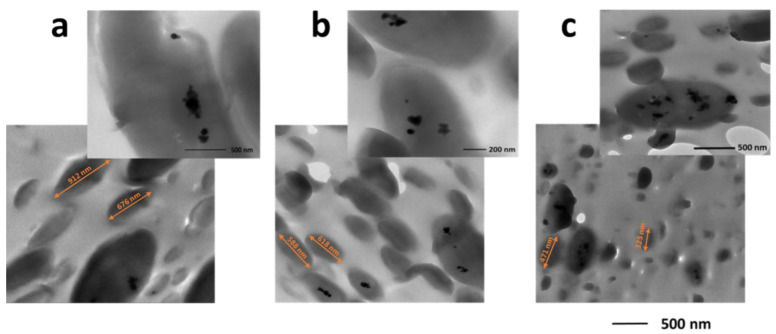
TEM images of the PLA/PA11 70/30 blends containing (**a**) 0.5% wt. ZnO, (**b**) 1% wt. ZnO and (**c**) 2% wt. ZnO.

The TEM images provided detailed information on the localization of the ZnO particles within the blends (Figure 4). For all the ZnO concentrations investigated, particle aggregates were mainly visible and were selectively localised within the polyamide droplets. Only at 2% wt. did some of them move to the interface between the two polymers (Figure 4c), thus exposing them to the PLA matrix. No particles were observed in the continuous phase, indicating a stronger affinity of ZnO to PA11. The localization of nanoparticles in immiscible polymer blends prepared by melt blending depends on the intrinsic physical parameters and processing conditions, including the blend components’ viscosities, temperature and mixing rate [14]. Interfacial tensions between the constituents play a crucial role in determining the wetting coefficient, $\omega_{a}$, which is usually used to predict the localization of nanoparticles in an immiscible polymer blend [47]. In our case, $\omega_{a}$ is defined as:

$$\omega_{a}=(\gamma_{ZnO-PLA}-\gamma_{ZnO-PA11})/\gamma_{PLA-PA11}$$

where $\gamma_{ZnO-PLA},\;\gamma_{ZnO-PA11}\;and$ $\gamma_{PLA-PA11}$ represent the interfacial tension between the ZnO and PLA, ZnO and PA11, and PLA and PA11, respectively. The interfacial tension values were calculated by using the geometric mean equation, as reported in the Supporting Information [48]. For $\omega_{a}$> 1, ZnO localization was expected in the PA11 dispersed phase; for $\omega_{a}$ < −1, localization would be expected in the PLA phase; and for 1 > $\omega_{a}$> −1, ZnO would be localised at the interface. The actual calculated $\omega_{a}$ value was 0.82, which supported preferential localization at the interface whilst indicating a stronger affinity of ZnO for the PA11 phase. The experimental evidence, which showed a preferential segregation of the ZnO particles in the polyamide phase, suggested that the used ZnO particles had a lower polarity than expected, which was likely due to the surface modification or to the formation of a compatibilising copolymer phase. The TEM analysis also showed a progressive reduction in the size of the PA 11 droplets upon increasing the ZnO content with a refinement of the phase morphology and the formation of a quasi-co-continuous blend. This outcome confirmed the compatibilization action due to the formation of a PLA/PA11 copolymer in the presence of higher quantities of ZnO, as emerged from the MALDI-TOF analysis. Moreover, the segregation of the filler inside the PA11 drops preserved the PLA matrix from nanoparticle-induced degradation, at least for the filler loadings low enough to not cause the protrusion of the nanoparticles from the host drops and their exposure toward the PLA phase.

## 4. Conclusions

PLA/PA11 immiscible blends filled with ZnO at different concentrations were formulated and studied by chemical, morphological and structural analyses. It was demonstrated that the addition of ZnO drastically modified the fate of the bioplastic blends prepared by melting extrusion. Indeed, up to a 1% addition of ZnO, the thermal properties increased, and the MMs did not change significantly during the preparation at 200 °C. Additionally, the presence of ZnO catalysed the trans-ester–amide reactions by forming PLA/PA11 block copolymers at the interface. With the addition of 2% of filler, a dramatic refinement of the PLA/PA11 blend morphology occurred, resulting in a compatibilised structure with a quasi-co-continuous phase.

Nevertheless, degradation reactions were also promoted for the higher amounts of ZnO, causing a drop in the MMs. ZnO addition, regardless of the %, accelerated UV photo-oxidative degradation for both the PLA and PA11 matrices due to the occurrence of well-known H abstraction and Norrish I and II photo-cleavages. Among these, the intervention of photocatalytic processes triggered by wurtzite with the formation of ROS molecules cannot be excluded. Finally, the formulations were subjected to natural aging by immersing them in artificial seawater. It was demonstrated that the addition of 0.5% wt. of ZnO to the PLA was enough to trigger its degradation in water media. This finding deserves further investigation since it is relevant from the perspective of the more sustainable exploitation of bioplastic materials.

## Figures and Tables

**Table 1:** Temperatures of main decomposition steps and residual masses at 700 °C.

	T_max_ (°C)	T_max_2 (°C)	T_5%_^a^	Residue at 700 °C (%/°C)
**PA11**	425		381.3	0.00
**PA11_0.5 ZnO**	455		397.2	0.52
**PA11_1 ZnO**	453		414.2	1.04
**PA11_2 ZnO**	445		397.6	2.9
				
**PLA**	377		256.9	0.19
**PLA_0.5 ZnO**	281		242.5	0.39
**PLA_1 ZnO**	291		239.4	1.42
**PLA_2 ZnO**	292		240.4	2.23
				
**PLA/PA11**	335.0	441.5	312.5	0.26
**PLA/PA11_0.5 ZnO**	350.9	453.3	301.2	0.83
**PLA/PA11_1 ZnO**	356.8	457.4	330.3	1.17
**PLA/PA11_2 ZnO**	278.5	456.8	241.7	3.72

**^a^** Temperature at a 5% weight loss.

**Table 2:** Average molecular weight (M_w_) values of PLA samples with different contents of ZnO nanofiller after immersion in artificial seawater and the % decrease in ΔM_w_(%), depending on ZnO amount.

Sample	M_w_^a^	PD ^b^	ΔM_w_ (%) ^c^
**PLA**	91,100	1.61	4^41^
**PLA@ZnO 0.5**	51,200	1.87	43
**PLA@ZnO 1**	50,200	1.87	44
**PLA@ZnO 2**	42,000	1.87	53

^a^ Mw of PLA samples with different contents of ZnO after immersion in artificial seawater for two weeks. ^b^ polydispersity index; ^c^ Mw decrease (%) of PLA samples with different contents of ZnO after immersion in artificial seawater for two weeks.

**Table 3:** Mw values of the THF soluble fraction of the PLA/PA11 nanocomposites after immersion in artificial seawater for two weeks.

Sample	M_w_ ^a^	PD ^b^	ΔM_w_ (%) ^c^
**PLA/PA11**	87,800	1.23	7
**PLA/PA11@ZnO_0.5**	58,200	1.42	33.7
**PLA/PA11@ZnO_1**	47,000	1.70	46.4
**PLA/PA11@ZnO_2**	26,400	1.99	69.9

^a^ M_w_ values of PLA/PA11 samples with different contents of ZnO after immersion in artificial seawater for two weeks; ^b^ polydispersity index; ^c^ M_w_ decrease (%) of PLA/PA11 samples with different contents of ZnO after immersion in artificial seawater for two weeks.

## Data Availability

Not applicable.

## Supplementary Materials

The following supporting information can be downloaded at: https://www.mdpi.com/article/10.3390/polym15061434/s1, Figure S1. SEM images of as purchased ZnO nanoparticles at different magnifications; Figure S2. MALDI-TOF spectrum of pristine PA11 sample registered in positive and reflectron mode; Figure S3. An enlarged portion from 1450–1700 m/z of MALDI-TOF spectrum of PA11 sample; Figure S4. MALDI-TOF spectrum of PA11 with 0.5% of ZnO; Figure S5. Detail of the MALDI-TOF spectrum of PA11@0.5 ZnO; Figure S6. MALDI-TOF spectrum of PA11 with 1% of ZnO; Figure S7. Detail of the MALDI-TOF spectrum of PA11@ZnO_1; Figure S8. MALDI-TOF spectrum of PA11@ZnO_2; Figure S9. Detail of the MALDI-TOF spectrum of PA11@ZnO_2; Figure S10. MALDI-TOF spectrum of pristine PLA; Figure S11. Detail of the MALDI-TOF spectrum of pristine PLA; Figure S12. MALDI-TOF spectrum of PLA@ZnO_0.5; Figure S13. Detail of the MALDI-TOF spectrum of PLA@ZnO_0.5; Figure S14. MALDI-TOF spectrum of PLA@ZnO_1; Figure S15. Detail of the MALDI-TOF spectrum of PLA@ZnO_1; Figure S16. MALDI-TOF spectrum of PLA@ZnO_2; Figure S17. Detail of the MALDI-TOF spectrum of PLA@ZnO_2; Figure S18. MALDI-TOF spectrum of the pristine blend PLA/PA11@ZnO_0.5; Figure S19. Detail of the MALDI-TOF spectrum of the pristine blend PLA/ PA11@ZnO_0.5; Figure S20. MALDI-TOF spectrum of PA11@ZnO_0.5 after the aging process (exposure to UV light, wavelength 340 nm, 168 h); Figure S21. MALDI-TOF spectrum of PLA@ZnO_0.5 after the aging process (exposure to UV light, wavelength 340 nm, 366 h); Figure S22. SEC profiles of PA11, PLA, and PLA/PA11 blend with and without ZnO addition; Figure S23. Distribution of PA11 droplets diameter with calculated mean and standard deviation for PLA/PA11, 140 PLA/PA11@ZnO_0.5; Figure S24. Overlap of the TGA profiles of PLA samples; Figure S25. Overlap of the TGA profiles of PA11 samples; Figure S26. Overlap of the TGA profiles of PLA/PA11 samples; Figure S27. Chromatograms of PLA samples with different amount of ZnO; Figure S28. Chromatograms of PLA/PA11 samples with different amount of ZnO. Table S1. main structures identified in the MALDI spectra; Table S2. Size distribution of PA11 droplets in the PLA/PA11 blends; Table S3. Surface tension, dispersive, and polar components (mJ/m2) for PLA, PA11, ZnO nanoparticles. References [15,47,49] are cited in Supplementary Materials File.
