# Supplementary material for: Photo- and Water-Degradation Phenomena of ZnO Bio-Blend Based on Poly(lactic acid) and Polyamide 11
Source: Polymers (Basel). 2023 Mar 14;15(6):1434. doi: 10.3390/polym15061434 (PMC10058673; doi:10.3390/polym15061434)
Supplement: Supplementary file 1 [file polymers-15-01434-s001.zip › polymers-2241627-supplementary.pdf]

polymers

Article – Supporting information

Photo and Water-Degradation Phenomena of ZnO Bio-Blend Based on Poly(Lactic Acid) and Polyamide 11

Roberta Puglisi [1], Andrea Antonino Scamporrino [1,*], Nadka Tzankova Dintcheva [2], Giovanni Filippone [3], Elena Bruno [4,5], Paola Scarfato [6], Pierfrancesco Cerruti [7] and Sabrina Carola Carroccio [1]

[1] Institute for Polymers, Composites and Biomaterials IPCB-CNR, Via P. Gaifami 18, 95126 Catania, Italy
[2] Dipartimento di Ingegneria, Università di Palermo, Viale delle Scienze, ed. 6, 90128 Palermo, Italy
[3] Department of Chemical, Materials and Production Engineering, University of Naples Federico II, Piazzale V. Tecchio 80, 80125 Naples, Italy
[4] Department of Physics and Astronomy "E. Majorana", University of Catania, Via S. Sofia 64, 95123 Catania, Italy
[5] Institute for Microelectronics and Microsystems IMM-CNR, Via S. Sofia 64, 95123 Catania, Italy
[6] Department of Industrial Engineering, University of Salerno, Via Giovanni Paolo II, 84084 Fisciano, Italy
[7] Institute for Polymers, Composites and Biomaterials (IPCB-CNR), Via Campi Flegrei 34, 80078 Pozzuoli, Italy
* Correspondence: andreaantonio.scamporrino@cnr.it; Tel.: +39-095-7338253

Citation: Puglisi, R.; Scamporrino, A.A.; Dintcheva, N.T.; Filippone, G.; Bruno, E.; Scarfato, P.; Cerruti, P.; Carroccio, S.C. Photo- and Water-Degradation Phenomena of ZnO Bio-Blend Based on Poly(Lactic Acid) and Polyamide 11. *Polymers* **2023**, *15*, 1434. https://doi.org/10.3390/polym15061434

Academic Editor: George Z. Papageorgiou

Received: 9 February 2023
Revised: 24 February 2023
Accepted: 8 March 2023
Published: 14 March 2023

Copyright: © 2023 by the authors. Submitted for possible open access publication under the terms and conditions of the Creative Commons Attribution (CC BY) license (https://creativecommons.org/licenses/by/4.0/).

Table S1

	Structures	*MNa+*	*MH+*
1	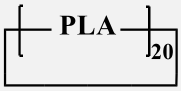	1463.40	
2	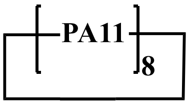	1488.26	1466.28
3	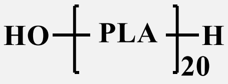	1481.39	
4	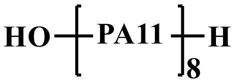	1484.27	1506.25
5	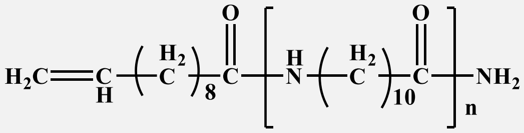	1488.36	1466.27
6	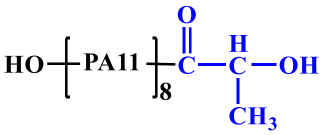	1578.68	
7	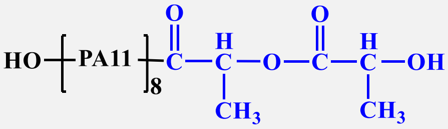	1650.78	

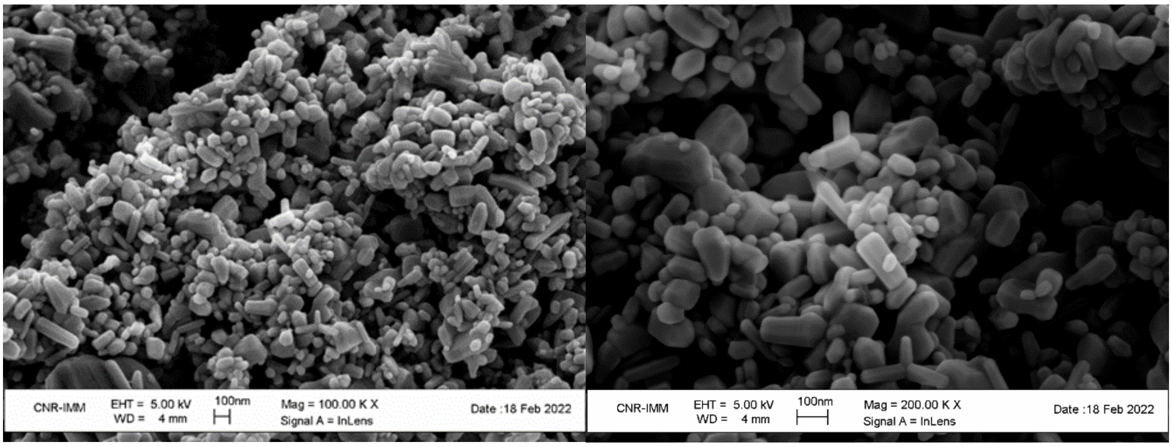

Figure S1. SEM images of as purchased ZnO nanoparticles at different magnifications

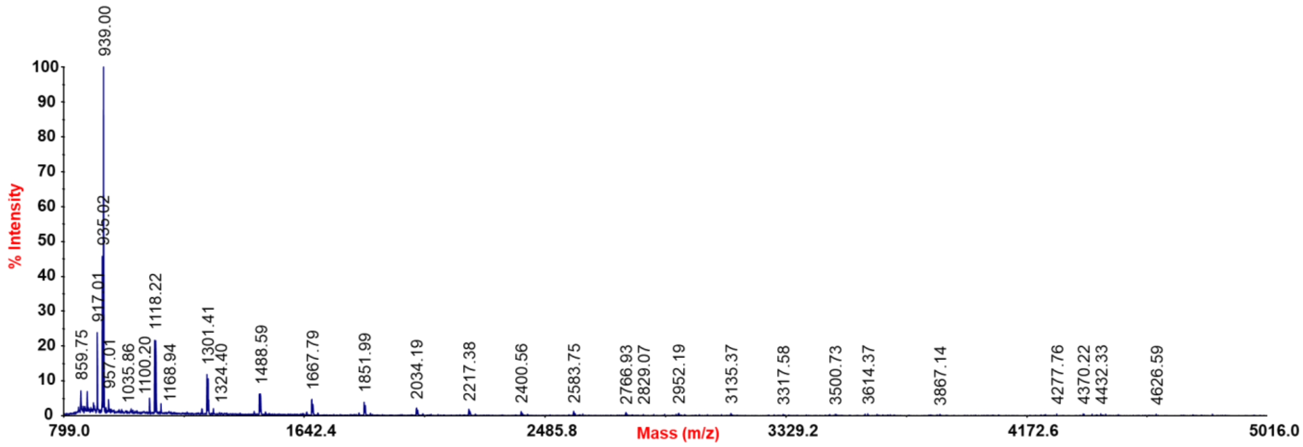

Figure S2. MALDI-TOF spectrum of pristine PA11 sample registered in positive and reflectron mode.

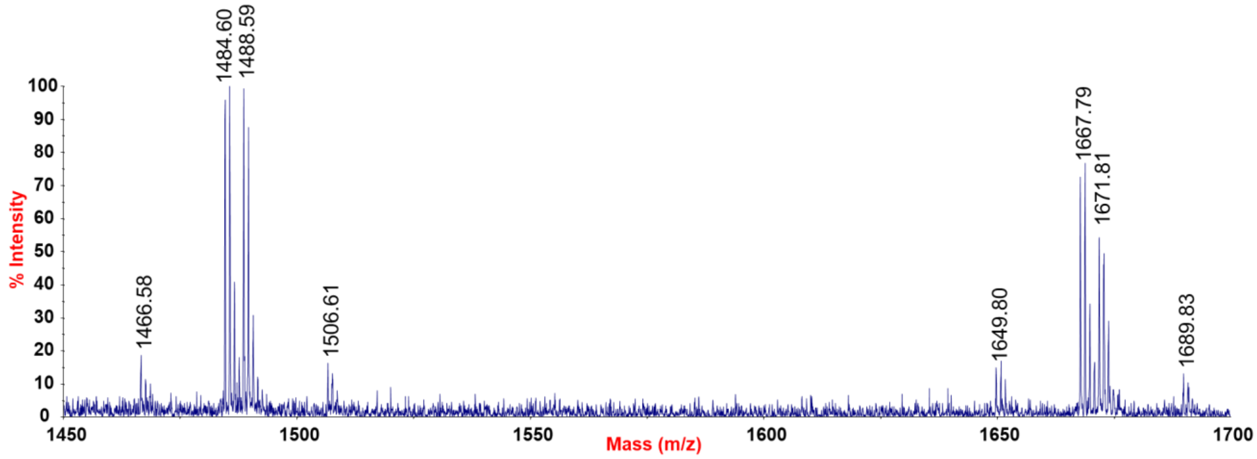

Figure S3. An enlarged portion from 1450-1700 m/z of MALDI-TOF spectrum of PA11 sample

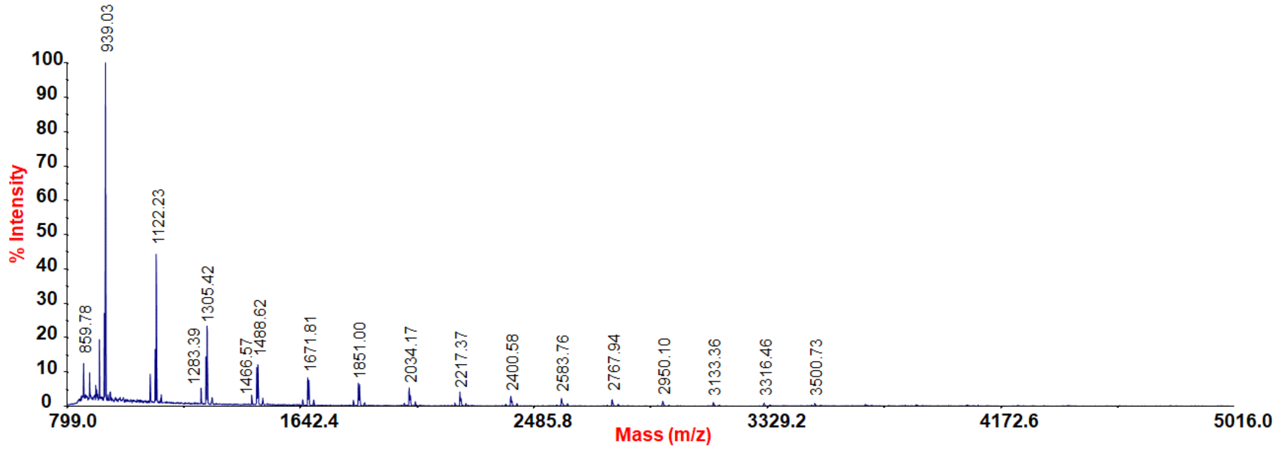

Figure S4. MALDI-TOF spectrum of PA11 with 0.5% of ZnO.

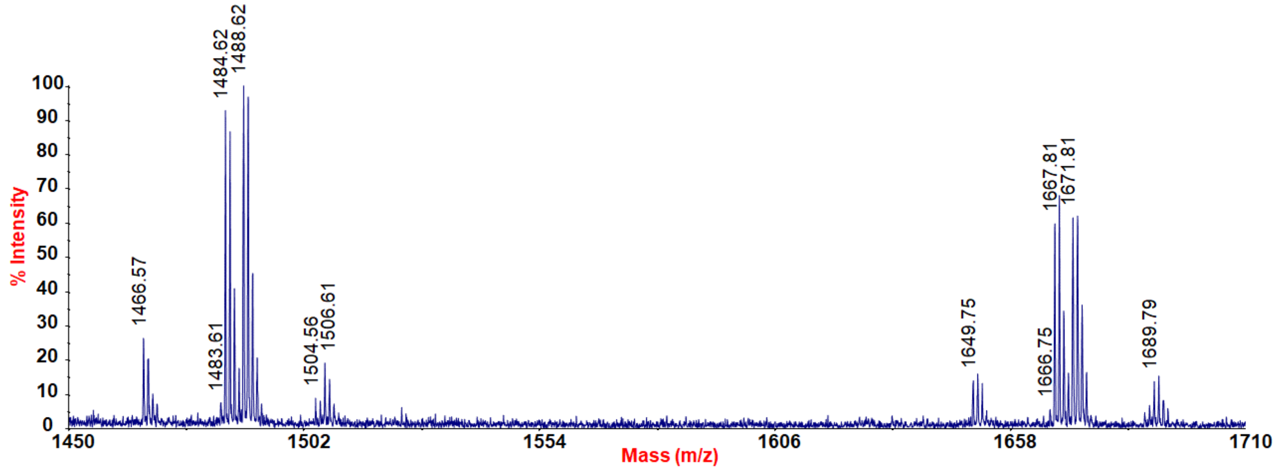

Figure S5. Detail of the MALDI-TOF spectrum of PA11@0.5 ZnO.

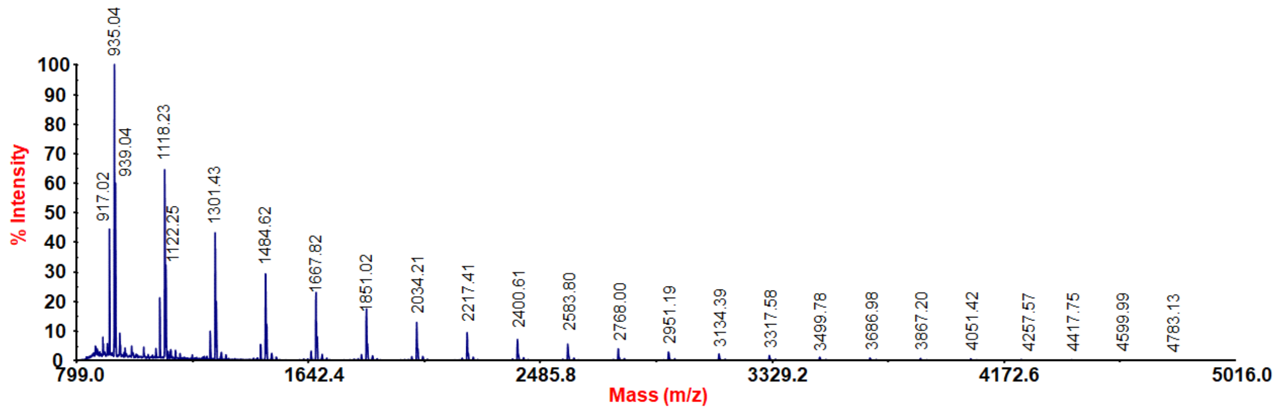

Figure S6. MALDI-TOF spectrum of PA11 with 1% of ZnO.

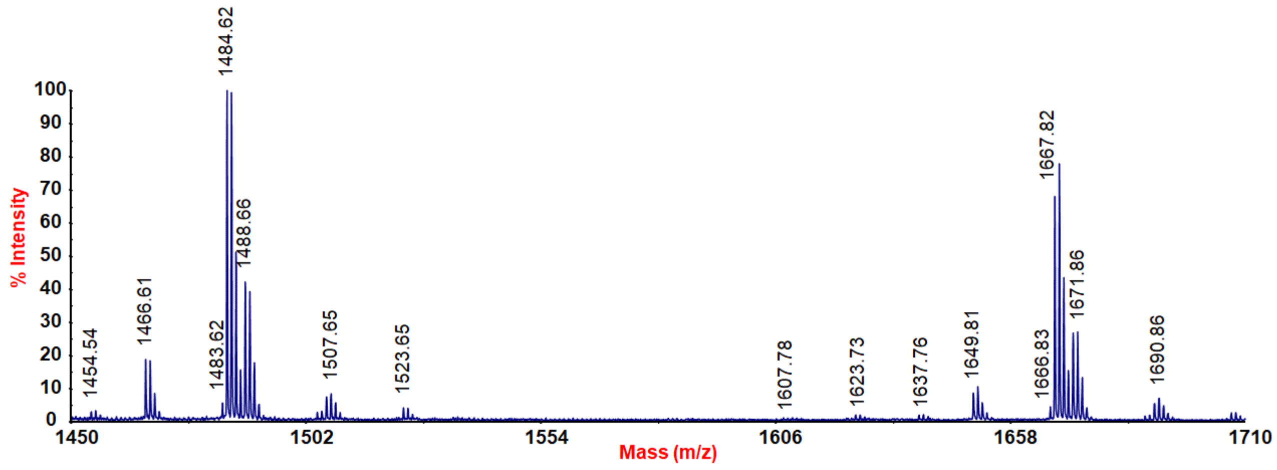

Figure S7. Detail of the MALDI-TOF spectrum of PA11@ZnO_1

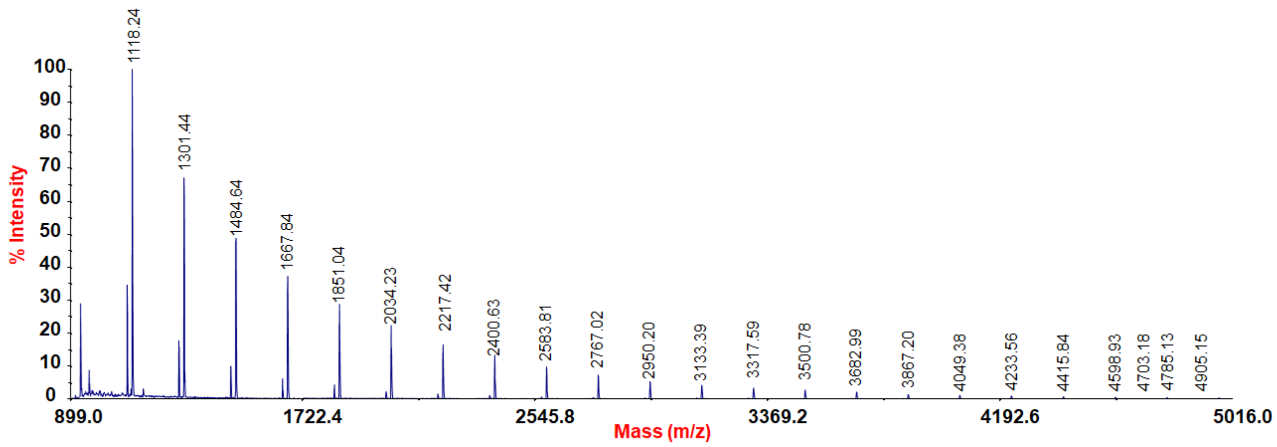

Figure S8. MALDI-TOF spectrum of PA11@ZnO_2.

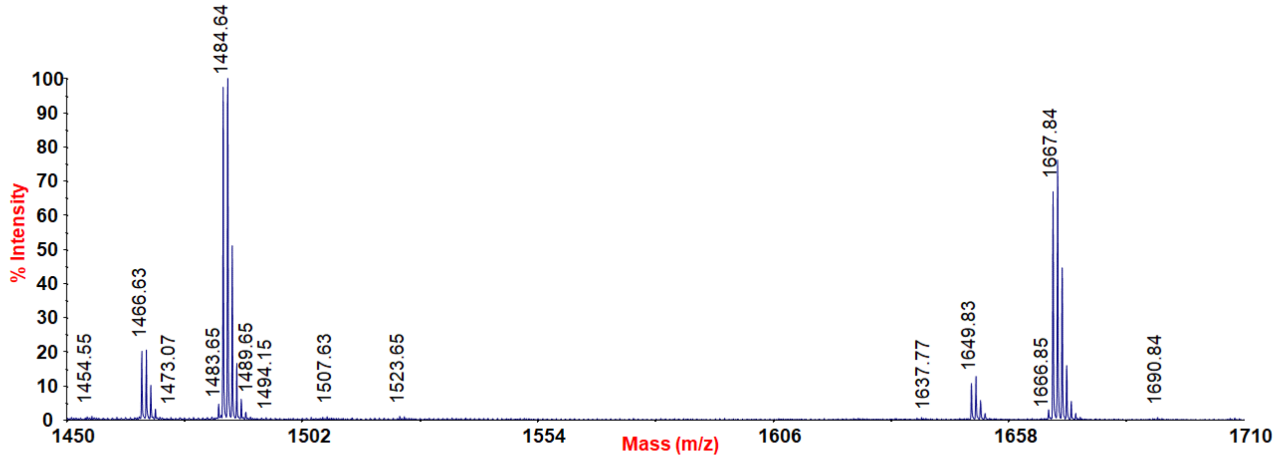

Figure S9. Detail of the MALDI-TOF spectrum of PA11@ZnO_2

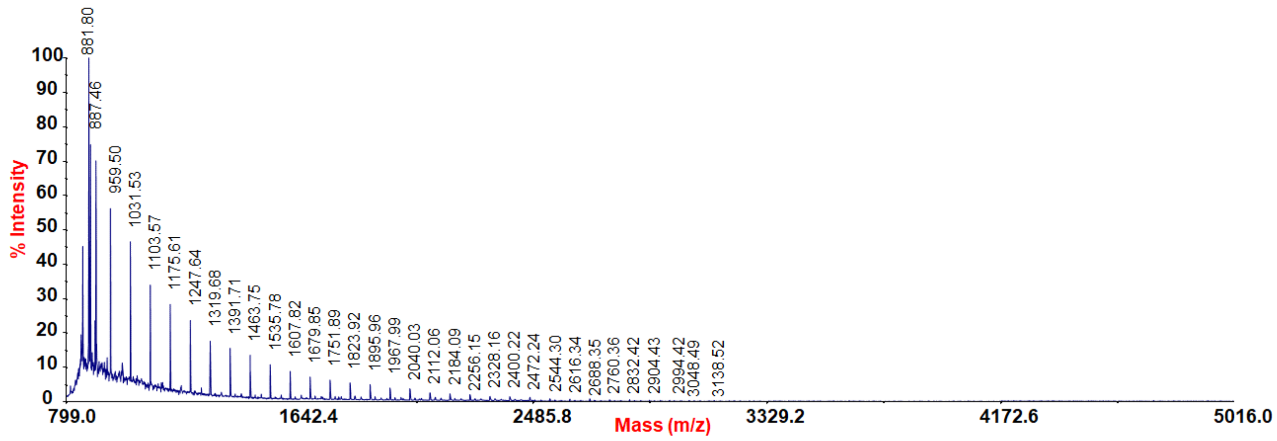

Figure S10. MALDI-TOF spectrum of pristine PLA.

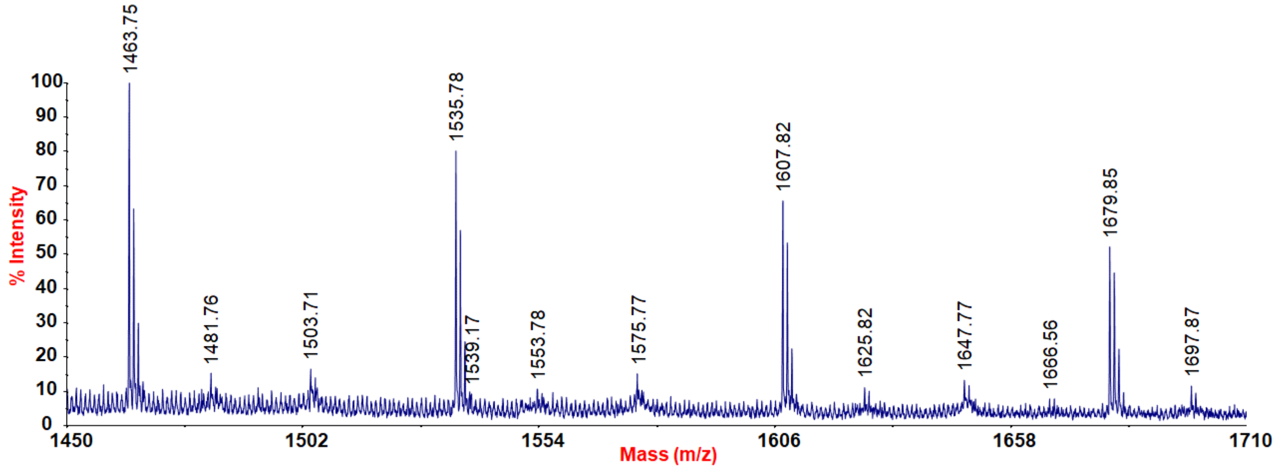

Figure S11. Detail of the MALDI-TOF spectrum of pristine PLA.

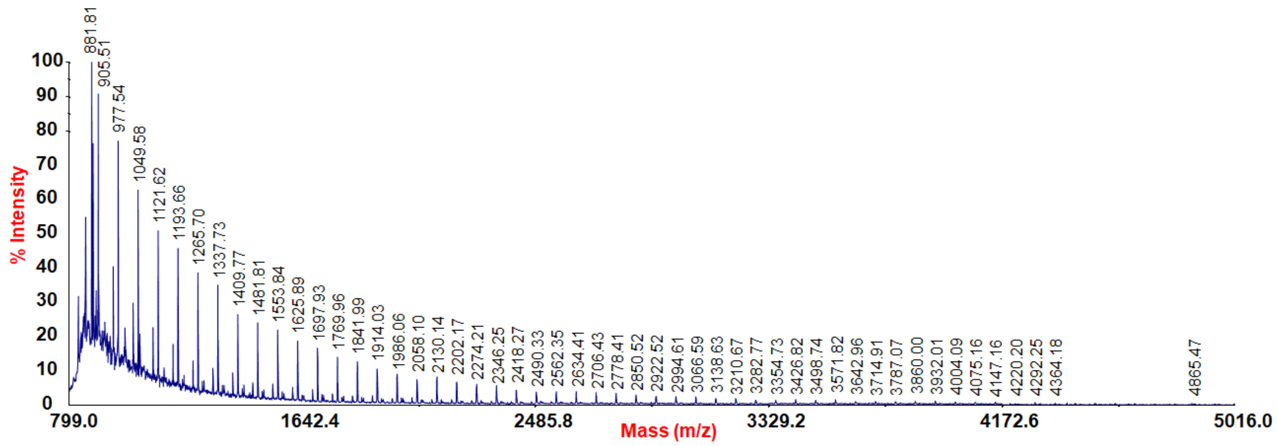

Figure S12. MALDI-TOF spectrum of PLA@ZnO_0.5.

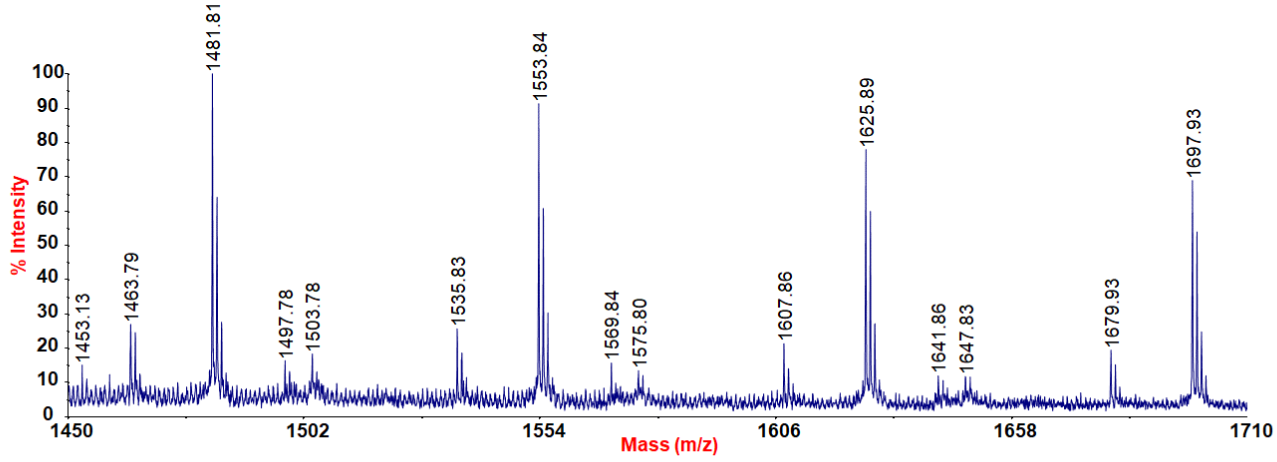

Figure S13. Detail of the MALDI-TOF spectrum of PLA@ZnO_0.5

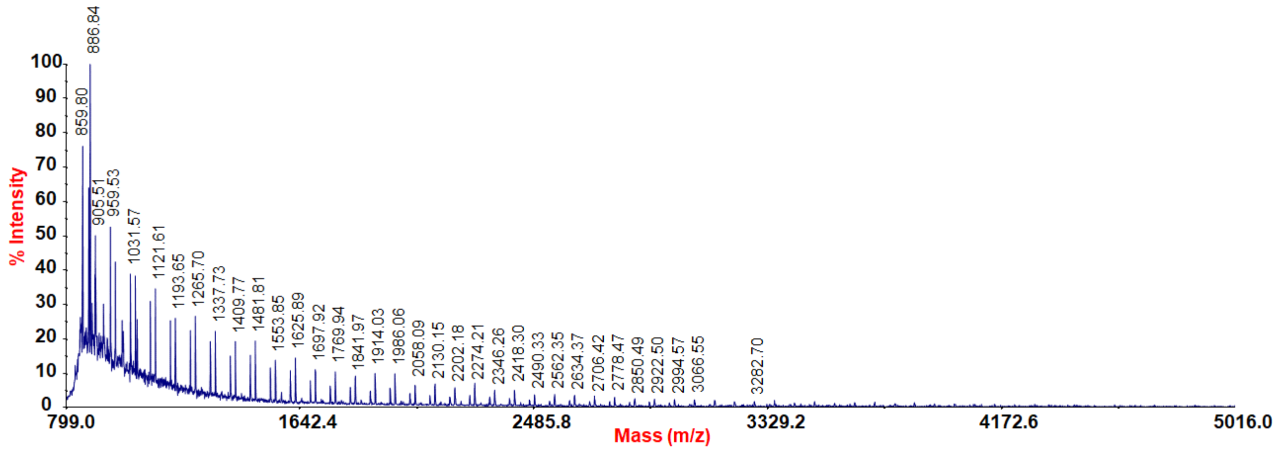

Figure S14. MALDI-TOF spectrum of PLA@ZnO_1.

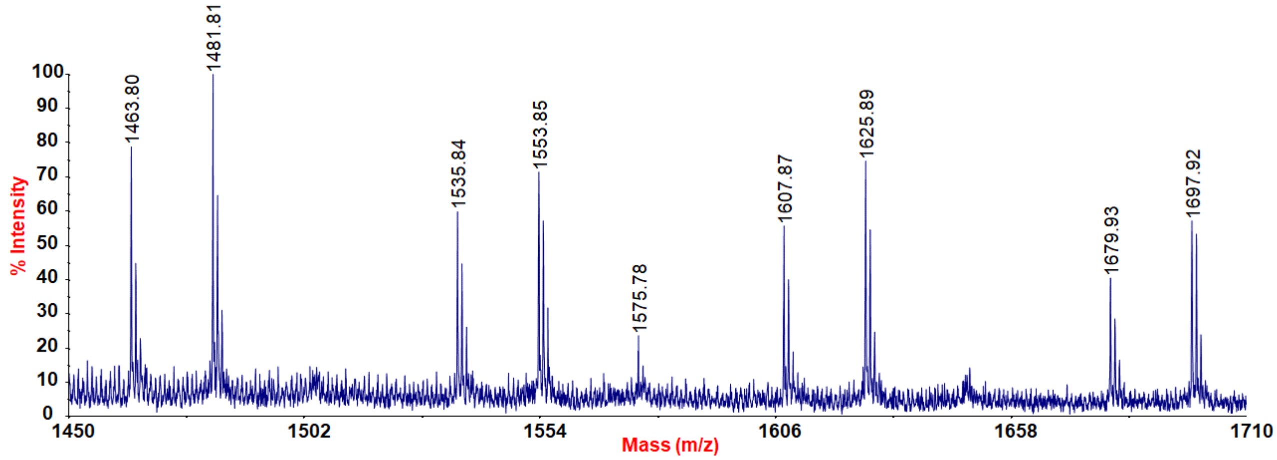

Figure S15. Detail of the MALDI-TOF spectrum of PLA@ZnO_1.

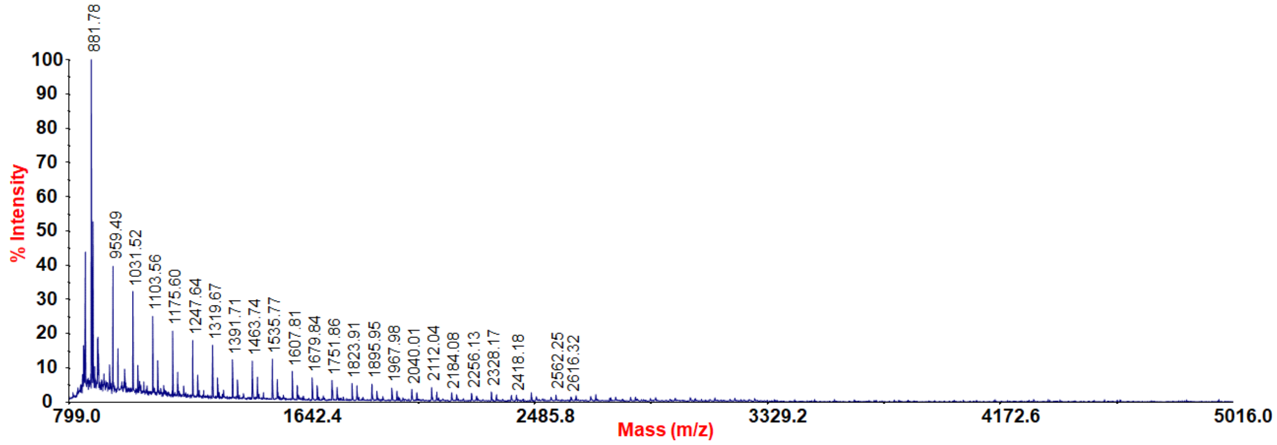

Figure S16. MALDI-TOF spectrum of PLA@ZnO_2.

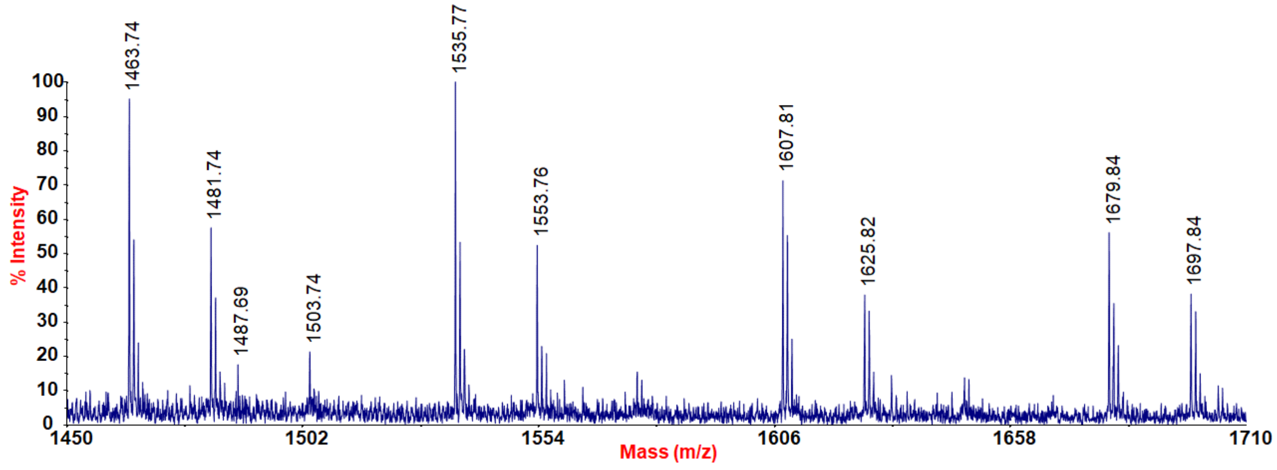

Figure S17. Detail of the MALDI-TOF spectrum of PLA@ZnO_2

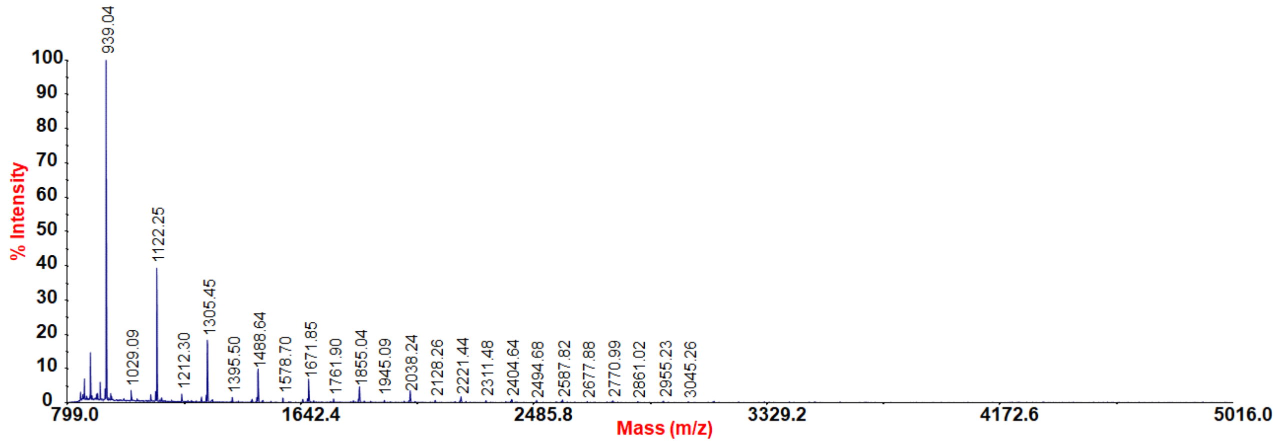

Figure S18. MALDI-TOF spectrum of the pristine blend PLA/PA11@ZnO_0.5

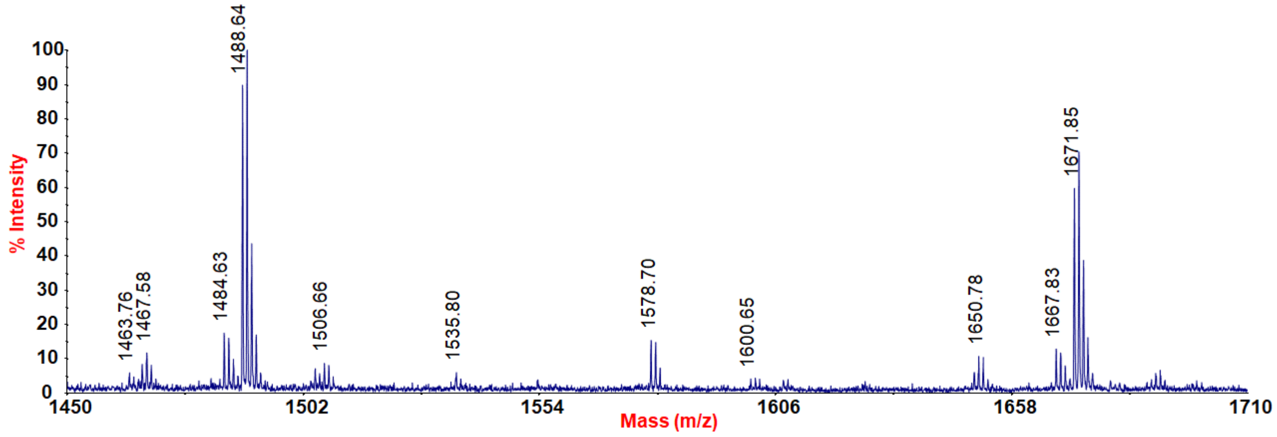

Figure S19. Detail of the MALDI-TOF spectrum of the pristine blend PLA/ PA11@ZnO_0.5

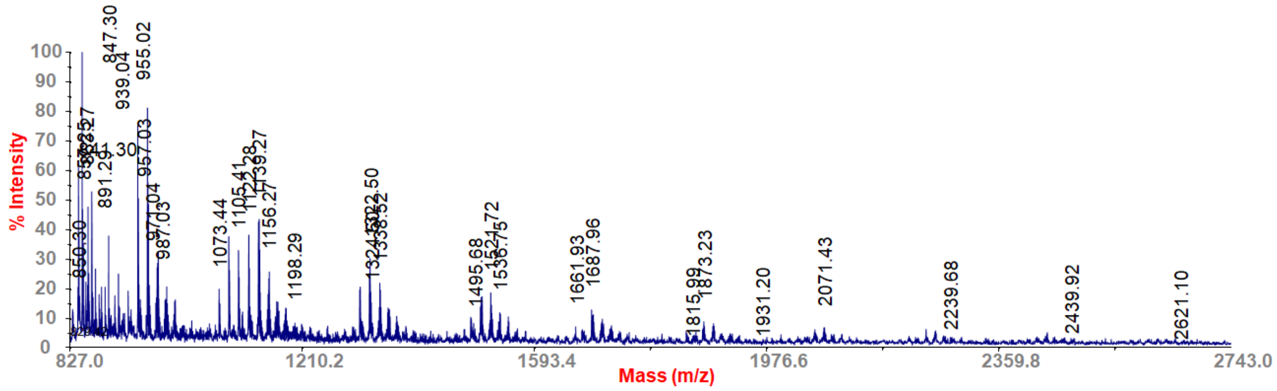

Figure S20. MALDI-TOF spectrum of PA11@ZnO_0.5 after the aging process (exposure to UV light, wavelength 340nm, 168 hours).

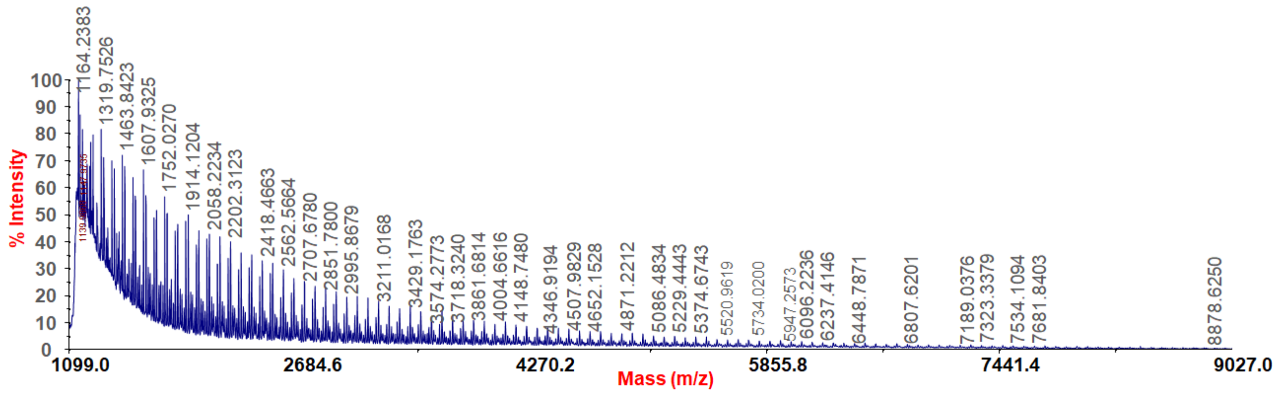

Figure S21. MALDI-TOF spectrum of PLA@ZnO_0.5 after the aging process (exposure to UV light, wavelength 340nm, 366 hours)

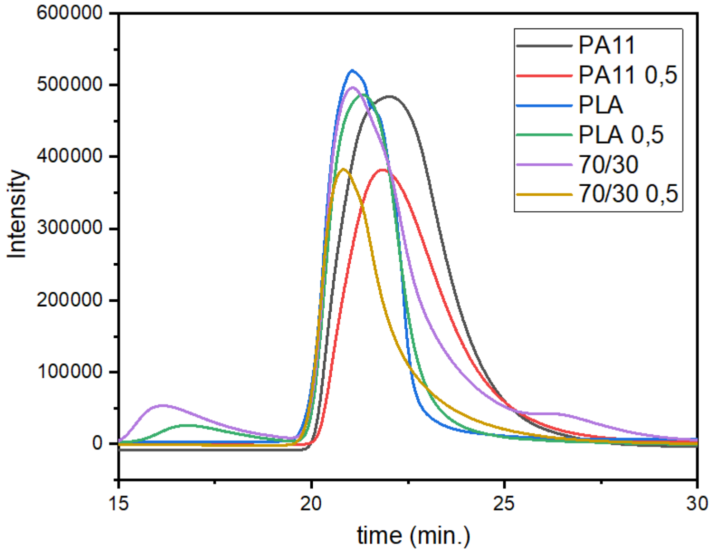

Figure S22. SEC profiles of PA11, PLA, and PLA/PA11 blend with and without ZnO addition

Table S2. Size distribution of PA11 droplets in the PLA/PA11 blends.

**Samples**	**Σni**[a]	d_n [μm][b]	***D***[c]
**PLA70/30**	204	1.02	1.36
**PLA70 ZnO 0.5%**	166	1.35	1.41
**PLA70 ZnO 1%**	155	1.43	1.13

[a]number of droplets analysed; [b]$(\Sigma_i n_i d_i)/(\Sigma_i n_i)$; [c]dv/dn

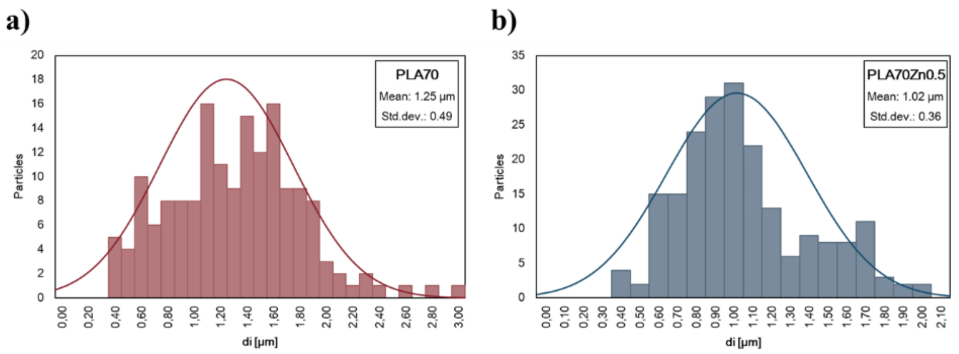

Figure S23. Distribution of PA11 droplets diameter with calculated mean and standard deviation for PLA/PA11, PLA/PA11@ZnO_0.5.

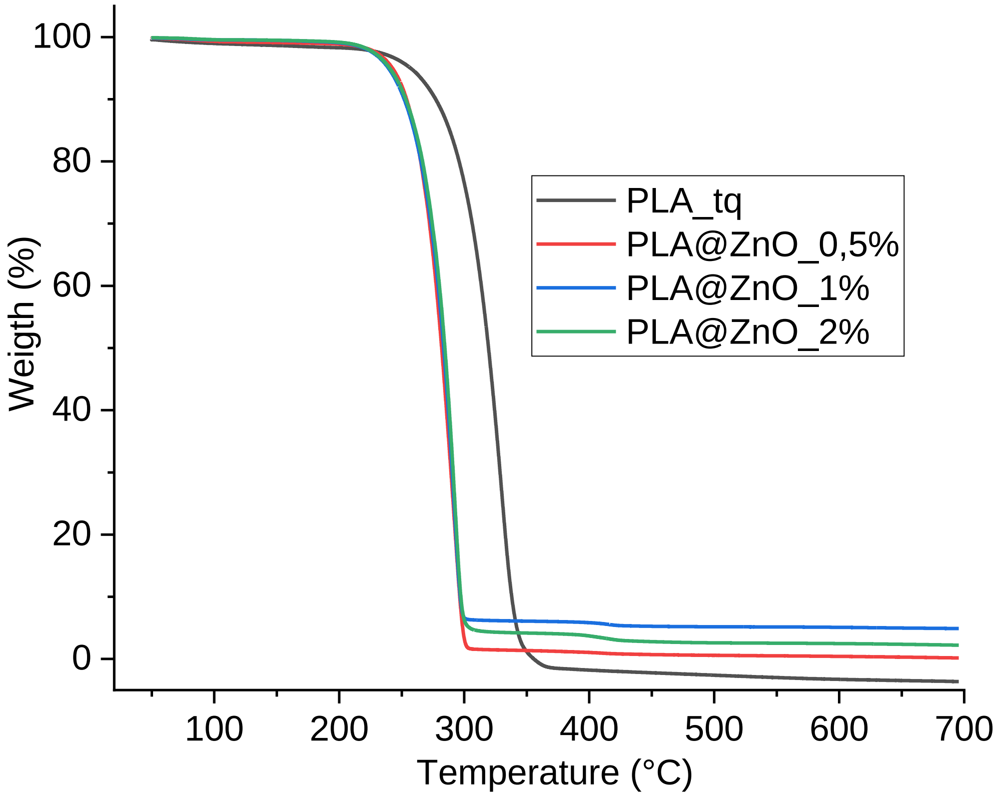

Figure S24. Overlap of the TGA profiles of **PLA** samples

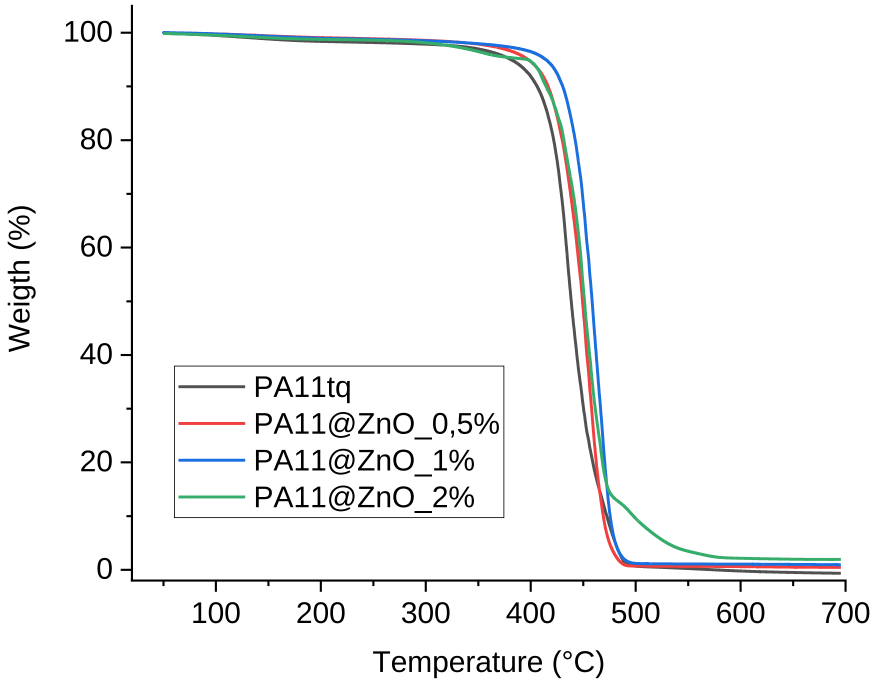

Figure S25. Overlap of the TGA profiles of **PA11** samples

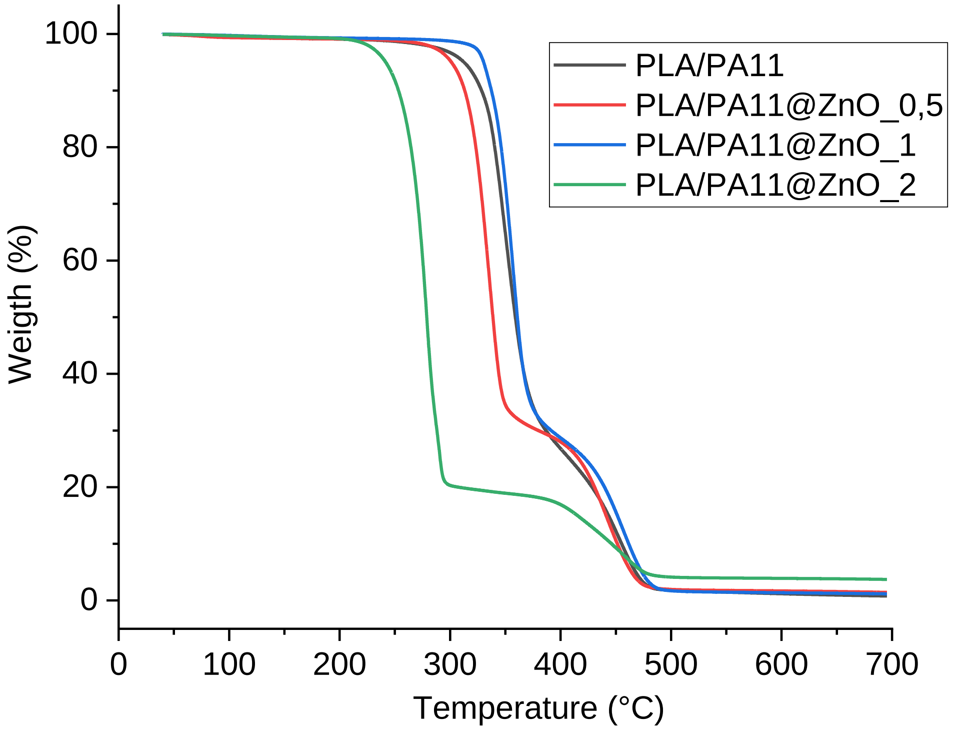

Figure S26. Overlap of the TGA profiles of **PLA/PA11** samples

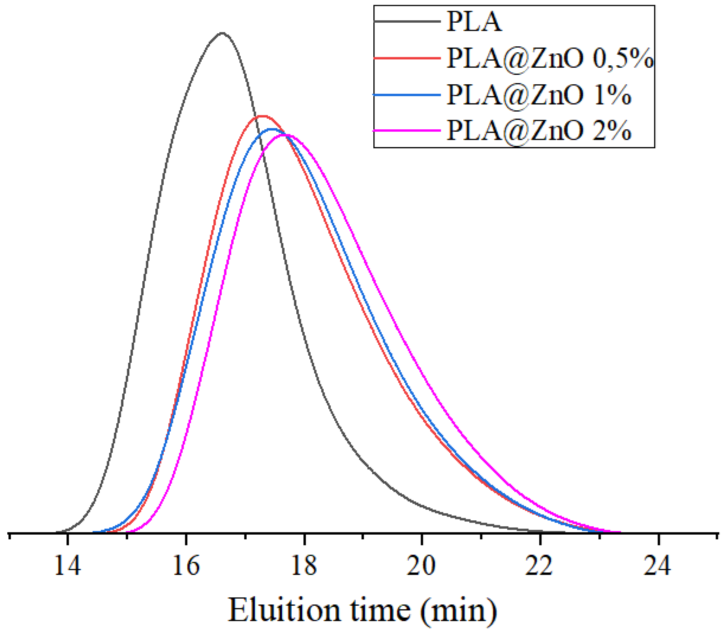

Figure S27. Chromatograms of PLA samples with different amount of ZnO.

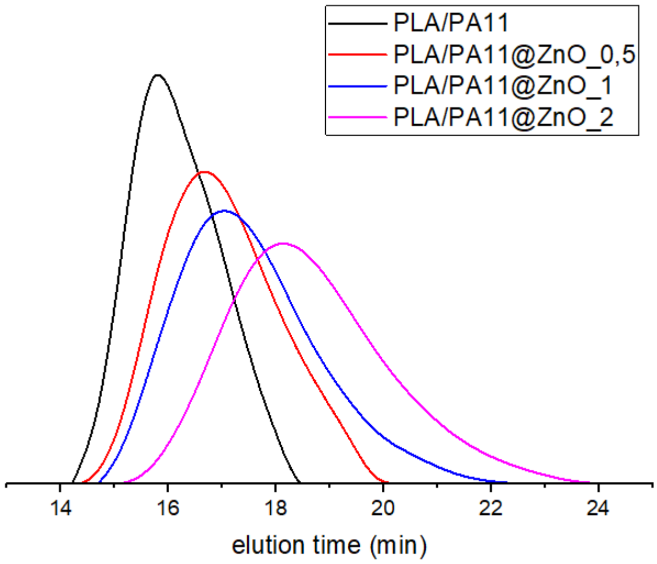

Figure S28. Chromatograms of **PLA/PA11** samples with different amount of ZnO.

Table S3. Surface tension, dispersive, and polar components (mJ/m^2) for PLA, PA11, ZnO nanoparticles.

Sample	γ	γ^d	γ^p
**PLA [1]**	38.5	7.9	30.5
**PA11 [1]**	41.8	31.4	10.4
**ZnO [2]**	34	25	9

The surface tension values listed in Table S3 were used to calculate the interfacial tension values between the blend components, using the Geometric-mean equation [3]:

$$\gamma_{ij} = \gamma_i + \gamma_j - 2(\gamma_i^d\ \gamma_j^d)^{1/2} + 2(\gamma_i^p\ \gamma_j^p)^{1/2}$$

where γ_{ij} are the surface tensions of component i and component j, respectively. The so calculated values of $\gamma_{ZnO-PLA}$, $\gamma_{ZnO-PA11}$, $\gamma_{PLA-PA11}$ were 11.26, 0.41, and 13.18 mJ/m^2, respectively.

[1] Helal, E., Pottier, C., David, E., Fréchette, M., & Demarquette, N. R. (2018). Polyethylene/thermoplastic elastomer/Zinc Oxide nanocomposites for high voltage insulation applications: Dielectric, mechanical and rheological behavior. *European Polymer Journal*, *100*, 258-269.

[2] Nuzzo, A., Bilotti, E., Peijs, T., Acierno, D., & Filippone, G. (2014). Nanoparticle-induced co-continuity in immiscible polymer blends–A comparative study on bio-based PLA-PA11 blends filled with organoclay, sepiolite, and carbon nanotubes. *Polymer*, *55*(19), 4908-4919.

[3] Xiao-dong Qi, Jing-hui Yang, Nan Zhang, Ting Huang, Zuo-wan Zhou, Ines Kühnert, Petra Pötschke, Yong Wang, Selective localization of carbon nanotubes and its effect on the structure and properties of polymer blends, Progress in Polymer Science, 123, 2021, 101471, https://doi.org/10.1016/j.progpolymsci.2021.101471.
